# Automated cell cycle and cell size measurements for single-cell gene expression studies

**DOI:** 10.1186/s13104-018-3195-y

**Published:** 2018-02-01

**Authors:** Anissa Guillemin, Angélique Richard, Sandrine Gonin-Giraud, Olivier Gandrillon

**Affiliations:** 1 Laboratoire de biologie et modélisation de la cellule. LBMC-Ecole Normale Supérieure-Lyon, Université Claude Bernard Lyon 1, Institut National de la Santé et de la Recherche Médicale: U1210-Ecole Normale Supérieure de Lyon, Centre National de la Recherche Scientifique: UMR5239, 46 Allée d’Italie, 69007 Lyon, France; 2 Inria Dracula, 69100 Villeurbanne, France

**Keywords:** Cell size, Cell cycle, Gene expression, Single-cell transcriptomic

## Abstract

**Objectives:**

Recent rise of single-cell studies revealed the importance of understanding the role of cell-to-cell variability, especially at the transcriptomic level. One of the numerous sources of cell-to-cell variation in gene expression is the heterogeneity in cell proliferation state. In order to identify how cell cycle and cell size influences gene expression variability at the single-cell level, we provide an universal and automatic toxic-free label method, compatible with single-cell high-throughput RT-qPCR. The method consists of isolating cells after a double-stained, analyzing their morphological parameters and performing a transcriptomic analysis on the same identified cells.

**Results:**

This led to an unbiased gene expression analysis and could be also used for improving single-cell tracking and imaging when combined with cell isolation. As an application for this technique, we showed that cell-to-cell variability in chicken erythroid progenitors was negligibly influenced by cell size nor cell cycle.

**Electronic supplementary material:**

The online version of this article (10.1186/s13104-018-3195-y) contains supplementary material, which is available to authorized users.

## Introduction

It has been known for decades that isogenic cells can differ from each other in their molecular composition [1, 2]. The refinement of molecular techniques together with computational approaches has recently allowed to get a quantitative view on this cell-to-cell variability. This strongly highlighted the importance of understanding the causes in such variations, leading to a recent turning point in single-cell studies [3–6].

A leading source of cell-to-cell variability can be attributed to stochastic gene expression [7–9]. Numerous factors contribute to cell-to-cell variability such as reactions involving a low-copy number of molecules especially during transcription processes [5, 7, 10, 11] or differences in the internal states of a cell population such as cellular age or cell cycle stage. In litterature, we can find contradictory results regarding the influence of cell cycle and cell size on gene expression. Some studies argued that both of these morphological parameters affect gene expression variation [7, 12–17] whereas, others support that this impact is negligible [18–21].

Stochastic gene expression takes various biological meaning [22–24]. In a cell fate context, stochastic gene expression could drive cells into the differentiation process [25]. It has been shown that during the erythroid differentiation process, we can observe an increase in cell-to-cell variability among genes expression that may participate to the decision making process within differentiation [21].

Together, these information highlight the importance to precisely identify the sources of gene expression variability involved in these phenomena in order to understand their role, and to discard potential confounding effects.

Cell cycle variability can be identified and suppressed by fluorescent-labeling of cell cycle-specific genes, however this method requires genetical alteration of the investigated cells [26]. Other studies, based on computational approach, deconvolute the cell cycle variables in order to normalize their single-cell gene expression data. Most of them use cell cycle marker genes to train algorithms that can predict cell cycle stage of individual cells [14, 27, 28]. However, these genes have different function or timing according to cell type, even in a same organism [29].

In this article, we propose a more direct approach that consists in measuring morphological parameters in a high-throughput single-cell RT-qPCR study. Using a non-cytotoxic double-staining technique we measured automatically cell cycle phase and cell size of every single-cell isolated from T2EC, a primary chicken erythroid progenitor cells [30]. We demonstrated that the labelling had no detectable effects at the single-cell transcriptomic level in those primary progenitors, suggesting that this technique could be an useful tool for molecular single-cell based studies.

We finally showed that in our cellular system neither cell size nor cell cycle state could be deemed responsible for the cell-to-cell variation we observed, ruling out their potential confounding effects.

## Main text

### Methods

#### Cell culture

T2EC were extracted from bone marrow of 19 days-old SPAFAS white leghorn chickens embryos (INRA, Tours, France). The composition of the culture medium has been previously described [21].

#### Double-staining

Cells were incubated in their initial medium for 30 min with CFSE (5-(and 6)-carboxyfluorescein diacetate succinimidyl ester, Life Tech.) at 5 μM and Hoechst 33342 (Life Tech.) at 5 μg/mL at 37 °C in a tube protected from light. After 2 washings in phosphate-buffered saline (PBS, Life Tech.), cells were loaded in the C1 system (Fluidigm).

#### RT-qPCR at population level

Cell culture were washed with PBS 4 h after the double-staining. Total RNA was extracted using RNeasy Mini Kit (Qiagen).

Reverse transcription assays were performed using the Superscript III First-Strand Synthesis System (Invitrogen) for 500 ng of total RNA.

Real-time PCR was performed with SYBR Green PCR Kit (ClonTech) in the CFX96 real-time PCR system (Bio-rad). Specific primers were used to quantify the expression of genes [21].

#### RT-qPCR at single-cell level

- From cell isolation to pre-amplification Cells were diluted with C1 cell suspension reagent (Fluidigm) at a concentration of $4\,\times\,10^5$ cells/mL. This step was followed by a cell filtration in a cellular sieve (50 μm). Cells were loaded in the C1 IFC (5–10 μm trap size, Fluidigm). The C1 system performed the cell isolation and pictures were taken with 2 different lasers (UV laser providing excitation at $\sim$ 350 nm and another at $\sim$ 488 nm) using a PALM-STORM NIKON Microscope (CIQLE). Then, the microplate was back in the C1 system where lysis, reverse-transcription and pre-amplification was performed. Primers have been previously described [21]. cDNA were loaded in a classic 96 well plate and conserved at − 20 °C until the RT-qPCR.
- Biomark real-time PCR quantification of cDNA were performed using EvaGreen following the Fluidigm user guide available on their website. Each condition was loaded in parallel in the same microfluidic-based chip to avoid chip-to-chip technical variability. An IFC Controller HX performed the load of cDNA samples and primers from the inlets into the chip. The Biomark HD analyzed the chip according to the GE 96 × 96 PCR + Melt v2.pcl program. RNA spikes were used as positive control to validate the RT-qPCR experiment. From this outlet, the analysis software generated cycle of quantification values ($C_q$) for each reaction.

#### ImageJ analysis

Each image corresponding at each lasers used were analyzed following a previously described procedure [31]. We visually confirmed the capture for each well and extracted automatically morphological information using ImageJ. After checking that all cells were detected by the software, we run the measurement of cell area (CFSE), nucleus area and intensity (Hoechst). The cell-volume (2) was then calculated from area measurements (1) using these following formulae:

1 $$\begin{aligned} r=\sqrt{\frac{S}{\pi }} \end{aligned}$$

2 $$\begin{aligned} V=\frac{4}{3} \times \pi \times r^3 \end{aligned}$$

with r the radius of cell, S the area and V the cell volume in ${\upmu }{\text{m}}^3$.

#### Analysis of gene expression

For population RT-qPCR analysis, ratios of gene expression variation between conditions were calculated following this following formulae [32]. Because of its low variability between all conditions, HnRNP was used as referential gene in these analyses.

For single-cell RT-qPCR, raw $C_q$ data was then computed using R [33] via a specific script that was previously described [21]. Some genes were excluded from analyses due to the quality control during the RTqPCR. The output file comprising absolute values of mRNA was used as a template for all following analysis. Statistical non-parametric tests were performed: correlations between gene expression and cell morphological parameters were performed using spearman tests. Wilcoxon tests were used to compare gene expression between stained and unstained conditions. Each time, Bonferroni correction was applied to p-values for the use of multiple tests.

#### PCA

PCAs were performed using ade4 package [34]. PCA was centered (mean substraction) and normalized (dividing by the standard deviation). PCA was displayed according to PC1 and PC2, which are the first and second axis of the PCA respectively.

### Results

#### Cellular morphological automatic measuring

**Fig. 1:**
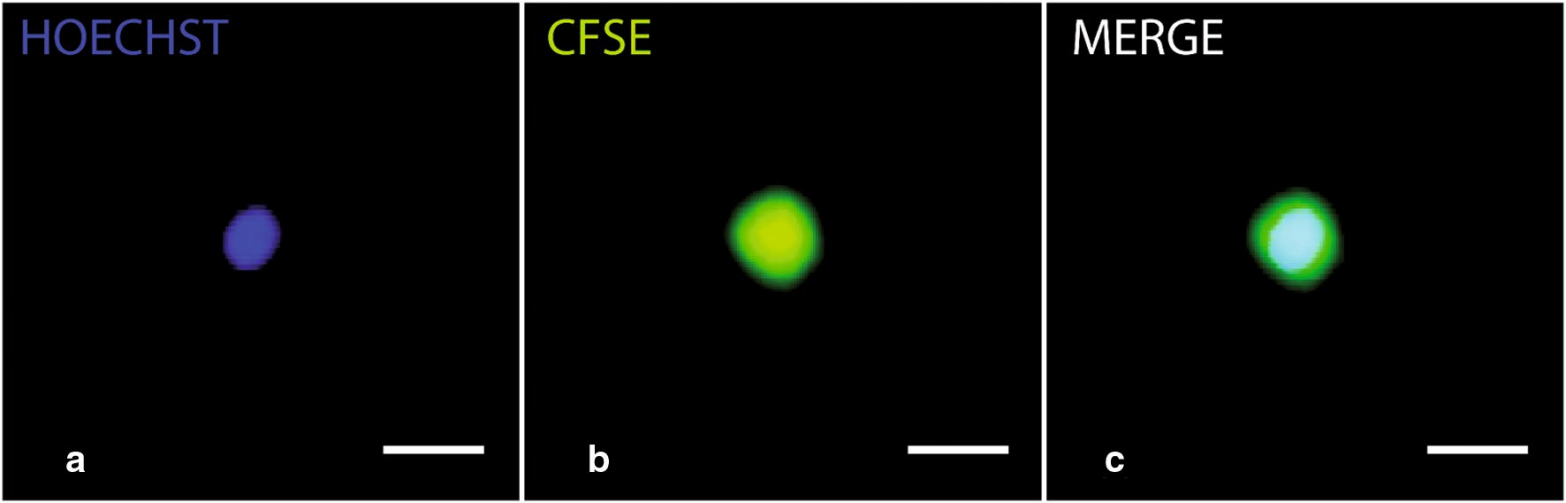
CFSE/Hoechst double staining is compatible with C1 technology. Typical labeling of T2EC nucleus (**a**) and cytoplasm/membrane (**b**) stained by Hoechst 33342 and CFSE respectively. **c** Merged image of **a**, **b**. Cells were isolated with the C1 system and observed using a Nikon microscope with 2 different lasers. The scale bar represents 10 μM

We choose the two low toxic fluorescent dyes, CFSE and Hoechst 33342 that stably incorporates into cells. In this study, CFSE was used as a cell area marker in tandem with Hoechst 33342 [35] as a nuclear marker. The use of two different lasers allowed revealing each staining (Fig. 1a, b) merged in Fig. 1c. It allowed us to automatically measure morphological cell parameters and inferred volumes.

**Fig. 2:**
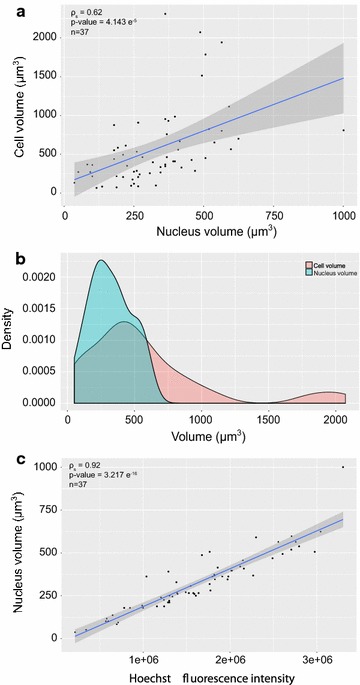
Analysis of cell and nucleus size measurements. **a** Scatter plot showing the relation between cell volume and nucleus volume. Each point represents a cell. Spearman correlation test was performed, the result of which is displayed in the left upper corner. **b** Distribution of cell volumes (red curve) and nucleus volumes (blue curve). **c** Scatter plot showing the relation between Hoechst fluorescence intensity and nucleus volume. Each point represents a cell. Spearman correlation test was performed, the result of which is displayed in the left upper corner

We can observe that the cell volume is very poorly correlated with the nucleus volume (Fig. 2a). Therefore cell size by itself does not seem to be a good proxy for determining cell cycle position probably because it integrated other unknown parameters. Both cell and nucleus volume density distributions confirm that cell size spans a much larger range than the nucleus size which displays the classical 2n/4n distribution (Fig. 2b). Nuclear-volume was clearly more correlated with Hoechst fluorescence intensity than cell-volume (Fig. 2a, c). The nucleus volume can therefore be considered as a good indicator for the position of the cell in the cell cycle. Furthermore it should be noted that volume is a purely geometrical object that is not influenced by the laser bleaching, as Hoechst fluorescence intensity parameter.

We therefore described a double-staining procedure compatible with microscopy associated at the C1 system to measure, for each cell, their size and cell cycle state independently.

#### Staining effect

**Fig. 3:**
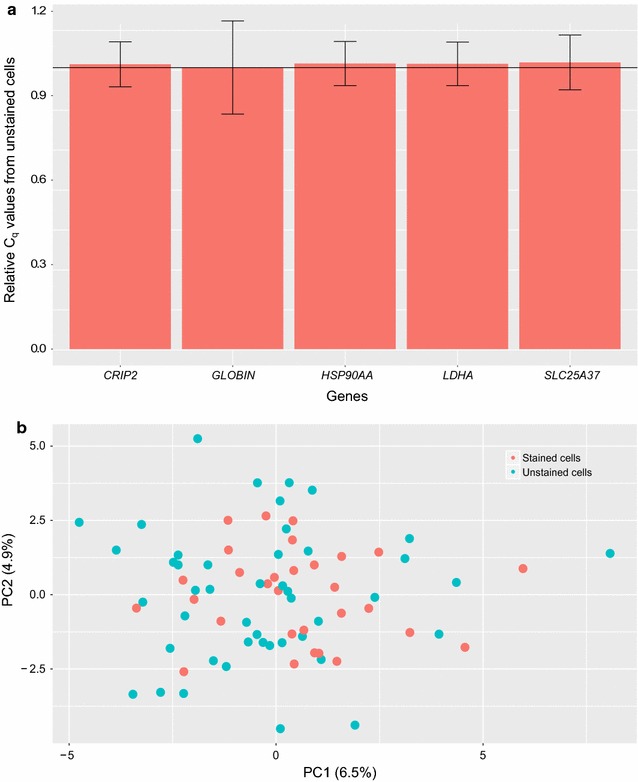
Analysis of the influence of the staining procedure on gene expression. **a** Real-time PCR gene expression analysis of stained and unstained cells. Total RNA was extracted from T2EC cells stained or not. Reverse transcription and real-time PCR analyses, with specific primers [21], were performed to quantify the amount of GLOBIN ($\beta$-GLOBIN), SLC (SLC25A37), HSP (HSP90AA1), CRIP2 and LDHA mRNA ($C_q$ for cycle of quantification). The fold variations represented here correspond to the ratio of mRNA of staining cells compared to unstained cells. The black line corresponds to the null variation between the two conditions. The vertical bars represent the standard error of the mean value (n = 3). **b** Principal Component Analysis of single cell expression data acquired on stained or unstained cells. Projection of 77 T2EC single-cell stained or not onto PC1 and PC2 results in a cloud of points without any clear separation. Percentages shown are the percentage of variance explained by each component

First, we assessed the influence of the double-staining procedure on gene expression at the population level by performing RT-qPCR on 5 selected genes known to be involved in erythroid differentiation or metabolism. The relative value of these gene expressions did not change significantly compared to unstained cells (Fig. 3a). These results suggested that cell and nucleus staining had no major influence on T2EC mean gene expression.

We then needed to discard possible modifications visible only at the individual-cell level. Therefore we performed high-throughput RT-qPCR on single cells using 77 genes that cover various functions as metabolism, differentiation process and proliferation [21]. We compared 30 single stained cells and 47 single unstained cells in the same microchip. Data was analyzed using a PCA-based dimensionality reduction algorithm (Fig. 3b) as well as Wilcoxon signed-rank tests (see Additional file 1: Table S1). The PCA does not show any separation between both conditions (PC1 and PC2 explained less than 12% of the variability), and the statistical analysis shows that no gene was significantly varying between the two conditions. These results therefore show that the staining did not affect the expression of these 77 genes in T2EC even when examined at the single-cell level.

Finally as an application example for our double-staining approach, we investigated the influence of cell cycle and cell size on cell-to-cell variability among our gene expressions using the coupling of labeling and gene expression measurements at the single-cell level.

#### Cell morphological impact on T2EC gene expression

For each single cell, we measured the size, the position in the cell cycle and the mRNA amount. Among 69 genes analyzed (retained in this study for technical quality control), none presented a significant spearman correlation between its expression level among single cell volumes or cell cycle: all p-values were above the 5% threshold. These results confirmed that neither cell size nor the position in cell cycle were relevant parameters in explaining the cell-to-cell variations observed for 69 genes examined. This information is important for stochastic single-cell-based gene expression analysis, for which these morphological parameters can be excluded of the potential sources of variability between cells.

### Conclusion

We performed a non-cytotoxic CFSE/Hoechst double-staining compatible with the C1 system. This approach allowed automatic identification and measure of morphological parameters. It can be used to measure the influence of cell cycle and cell size on single-cell gene expression analysis without any potential misleading cell state effects induced by cell cycle synchronization methods. It could be also represent an alternative method to avoid artificial cell sorting according to their size or their cell cycle phase, which could be interesting for low amount of cells. This is equivalent to the recently described technique using flow cytometry [36], but applicable in the C1 system. As an alternative, it has recently been described that predefined gene combination could be used a posteriori [37]. Unfortunately, the best combinations seems to be cell type dependent, making it potentially limited [38].

We then used the Biomark system to perform gene expression quantification. We showed that the double staining did not impact gene expression in our cells. Moreover, by measuring the influence of cell cycle and cell size on the expression level of 69 genes, our results support our previous claim that cell cycle and cell size have a negligible influence on gene expression variability in certain settings [21]. This is in line with the recent demonstration that the cell cycle explains only a very small amount (5–17%) of gene expression variability [18, 20].

## Limitations

In this study, the main limitation was the optimization of cell capture in the C1 microchip. We obtained a maximum of 65% of capture whereas with other cells, this percent raise up to 95%. Numerous parameters were involved and have to be optimized in order to obtain more individual cells per microchip.

## Additional file

**Additional file 1: Table S1.** Statistical analysis of gene expression according to the stained or the unstained condition. Statistical tests (Wilcoxon) were performed for each gene between their expression in stained and unstained condition. A Bonferroni correction was applied in p-values for multiple tests.

## Abbreviations

(RT-q)PCR: reverse transcription quantitative polymerase chain reaction
T2EC: TGF-α/TGF-β-induced erythrocytic cells
(c)DNA: complementary deoxyribonucleic acid
(m)RNA: messenger ribonucleic acid
CFSE: carboxyfluorescein diacetate succinimidyl ester
PBS: phosphate-buffered saline
HnRNP: heterogeneous nuclear ribonucleoprotein
GE: gene expression
PCA: Principal Component Analysis
PC1: first principal component
PC2: second principal component
