# Supplementary material for: Automated cell cycle and cell size measurements for single-cell gene expression studies
Source: BMC Res Notes. 2018 Feb 1;11:92. doi: 10.1186/s13104-018-3195-y (PMC5796519; doi:10.1186/s13104-018-3195-y)
Supplement: Supplementary file 1 — Additional file 1: Table S1. Statistical analysis of gene expression according to the stained or the unstained condition. Statistical tests (Wilcoxon) were performed for each gene between their expression in stained and unstained condition. A Bonferroni correction was applied in p-values for multiple tests. [file 13104_2018_3195_MOESM1_ESM.pdf]

Table 1: Statistical analysis of gene expression according to the stained or the unstained condition

Genes	p-values
AACS	1
ABCG2	1
ACSL6	1
ACSS1	1
ALAS1	1
ARHGEF2	1
BATF	1
BCL11A	1
HBB	1
BPI	1
CD151	1
CD44	1
CREG1	1
CRIP2	1
CTCF	1
CYP51A1	1
DCP1A	1
DCTD	1
DHCR24	1
DPP7	1
EGFR	1
EMB	1
FAM208B	1
FDFT1	1
FHL3	1
FNIP1	1
GAB1	0.722225076
GLRX5	1
GPT2	1
GSN	1
HMGCR	1
HMGCS1	0.260105866
HRAS1	1
HSD17B7	1
HSP90AA1	1
HYAL1	1
LCP1	1
LDHA	1
MAPK12	1
MFSD2B	1
MID2	1
MKNK2	1
MYO1G	1
NCOA4	1
NSDHL	1
PDLIM7	1
PIK3CG	1
PLAG1	1
PPP1R15B	1
PTPRC	1
RBM38	1
REXO2	1
RFFL	1
RHPN2	0.333534863
RPL22L1_1	1
RUNX2	1
sca2	1
SCD	1
SERPINI1	1
SLC25A37	1
SLC9A3R2	1
SMPD1	1
SNX22	0.769985306
SQLE	1
SQSTM1	0.846827928
STARD4	1
STX12	1
SULF2	1
SULT1E1	0.655470959
TADA2L	1
TBC1D7	1
TNFRSF21	1
TTYH2	1
UCK1	1
UNKNOWN8	1
VRK3	1
XPNPEP1	1
